# Structural Rheology of the Smectic Phase

**DOI:** 10.3390/ma7075146

**Published:** 2014-07-16

**Authors:** Shuji Fujii, Shigeyuki Komura, Chun-Yi David Lu

**Affiliations:** 1 Department of Chemistry, Nagaoka University of Technology, Nagaoka 940-2188, Japan; 2 Department of Materials and Interfaces, Weizmann Institute of Science, Rehovot 76100, Israel; 3 Department of Chemistry, Tokyo Metropolitan University, Tokyo 192-0397, Japan; E-Mail: komura@tmu.ac.jp; 4 Department of Chemistry, National Taiwan University, Taipei 106, Taiwan; E-Mail: cydlu@ntu.edu.tw

**Keywords:** soft matter, structural rheology, smectic phase, dislocation loop, focal conic domain, unbinding transition, lamellar phase, onion structure

## Abstract

In this review article, we discuss the rheological properties of the thermotropic smectic liquid crystal 8CB with focal conic domains (FCDs) from the viewpoint of structural rheology. It is known that the unbinding of the dislocation loops in the smectic phase drives the smectic-nematic transition. Here we discuss how the unbinding of the dislocation loops affects the evolution of the FCD size, linear and nonlinear rheological behaviors of the smectic phase. By studying the FCD formation from the perpendicularly oriented smectic layers, we also argue that dislocations play a key role in the structural development in layered systems. Furthermore, similarities in the rheological behavior between the FCDs in the smectic phase and the onion structures in the lyotropic lamellar phase suggest that these systems share a common physical origin for the elasticity.

## 1. Introduction

Rheology is a fundamental issue in soft matter science. One of the most successful achievements in the rheology of soft matter is the Doi-Edwards model, which describes the viscoelastic response of entangled polymer melts [1]. This model guides further theories and experiments which contribute not only to the industrial application of polymer materials but also to the progress of the basic polymer science. In contrast to the success in the polymer systems, the rheology of soft matter with meso-scale structures is still a developing field. Structured fluids such as foam, emulsions and colloidal systems as well as polymers have been also widely studied for many industrial applications [2–5]. However, the attempt to describe their universal rheological properties has only started using the concept of “soft glassy rheology” [5,6]. Besides these glassy materials, the rheology of surfactant systems which exhibit the gyroid phase with a three-dimensional periodic structure, or the sponge phase with randomly connected bicontinuous interface remains unexplored except for some pioneered studies [7–12]. Their unique viscoelastic responses arise predominantly from deformation of meso-scale internal structures whose rearrangement can be easily induced under deformation or flow.

The rheology of soft matter looks for a fundamental understanding in terms of the micro/meso structures of the systems. Representative examples of “structural rheology” include emulsions, foams, colloidal dispersions, surfactant solutions, and liquid crystals. Molecular systems such as a lubricant confined in a narrow space also exhibit various responses depending on their microstructure [13–15]. Since there are many different structures, to unify the rheology of structured fluids is an attractive yet challenging subject. Furthermore, as the soft structures can also evolve in the flow, a good selection of the structure unit is essential to understand the system. If we can establish a fundamental principle for the “structural rheology”, the significance of the soft matter in the industrial application will increase. In this review, we take defects as the key structures to understand the smectic rheology.

In this review article, we discuss the structural rheology of thermotropic smectic liquid crystal as a typical example [16–19]. Among various soft materials which spontaneously form internal structures such as lamellar, hexagonal, cubic, and gyroid phases [20–23], the simplest one-dimensional periodic structure is the smectic-A phase in thermotropic liquid crystals. The smectic liquid crystals exhibit a solid-like response in the layer perpendicular direction and a fluid-like response within the layers. Although the solid and the fluid-like responses do not mix for the smectic with a perfect alignment, self-organized textures combine these responses and give rise to the viscoelasticity. Even in such simple systems, phenomena such as shear-thinning and the orientation transition of the smectic layer are observed once a flow field is applied [24–29]. In lyotropic lamellar phases, it is known that bilayer membranes form multi-lamellar vesicles (onions) under shear flow [30–34].

As Horn and Kleman [35] pointed out in their pioneering work, smectic rheology is influenced by defects in the bulk. It has been explained that shear-thinning behavior depends on the defect dynamics and/or the defect density [25,36–38]. Although the importance of defects in the smectic rheology is a common understanding, there are relatively few studies that focus on the role of defects either explicitly or systematically [39–42]. Meyer *et al.* and Lu *et al.* [36–38] studied the shear-thinning behavior by considering the dynamics of screw dislocations and dislocation loops. They found that the theoretically predicted shear-thinning behavior *γ̇* ∼ *σ*^m^, where *γ̇* is the shear rate and *σ* the shear stress, was consistent with the experimental results within a limited range of the shear rate.

The thermotropic smectic liquid crystal 8CB changes from the crystalline phase to the smectic phase at *T* =21.5 °C, and further to the nematic phase at *T*_SN_ =33.4 °C. In this review article, using 8CB as a typical example of smectics, we summarize the structural observation, the linear and nonlinear rheological behavior of the smectic-A phase close to (but below) *T*_SN_. In the next section, we briefly explain the defects in the smectic liquid crystal phase. The temperature and the shear rate dependences of the defect size are discussed in Section 3. In Section 4, the nonlinear rheology of the smectic phase is investigated from the viewpoint of unbinding of dislocations, and summarize them in a dynamic phase diagram. The physical origin of the elasticity of the smectic phase with defects is suggested in Section 5. In the following section, we explain the dynamics of defect formation induced by a non-equilibrium phase transition in the smectic phase under flow. Finally, we mention the similarities between textural defects in the thermotropic smectic phase and the onion structures in the lyotropic lamellar phase.

In our study, we did not perform any surface treatment of the shear cell. The lack of the surface anchoring may induce the misalignment of the smectic layers and lead to the nucleation of focal conic domains (FCDs). However, in our experiment, reproducible results could be obtained by applying the pre-shear even without any anchoring treatment.

## 2. Defect Structures in the Smectic Phase

A liquid crystal has fluidity of a liquid and elasticity of a crystal. It also contains defects which locally break the translational symmetry and form reconnected layers [43]. Smectic-A phase with a layered structure is perturbed by two types of line defects; edge dislocations and screw dislocations which are parallel and perpendicular to the layer surface, respectively. These line defects appear in pairs with opposite signs so that two screw dislocations with opposite signs linked by edge dislocations form a dislocation loop. Since the dislocation loop locally compresses the layer thickness, an increase in the dislocation loop density causes the accumulation of strain energy. Furthermore, FCDs are formed to relax the compression deformation [44,45]. FCDs are visible under an optical microscope and have sizes from a few micrometers to a hundred micrometers.

**Figure 1.**
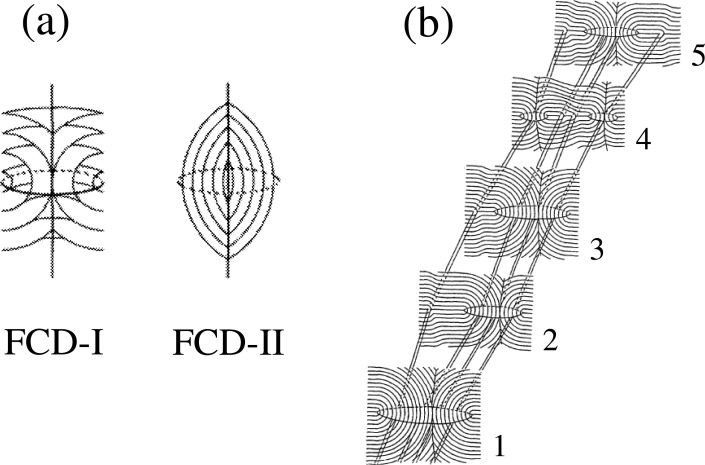
Schematic diagrams of (**a**) two different types of focal conic domains, FCD-I and FCD-II; and (**b**) oily streak structure in which five vertical cross sections of FCD-I are connected by four edge dislocations [45,46]. In the FCDs, the layers are folded into Dupin cyclides with ellipse and hyperbola so that the curved layers remain equidistant. Reproduced by permission of EDP sciences.

As shown in Figure 1, there are two types of FCDs which can be distinguished by the sign of the Gaussian curvature of the layers. The first type, FCD-I, has negative Gaussian curvature forming a toroidal shape, and is frequently observed in thermotropic liquid crystals. Whereas the second type, FCD-II, has a concentric sphere like onion structure which is observed only in lyotropic lamellar phases. As presented in Figure 1b, FCD-Is are connected by edge dislocations to form “oily streaks” which are commonly observed both in thermotropic and lyotropic systems [46].

Not only the edge dislocations but also the screw dislocations also affect the FCD structure. In the FCDs, the layers are folded into Dupin cyclides with ellipse and hyperbola which can be identified as disclinations. Kleman *et al.* [47] and Meyer *et al.* [48] experimentally observed that the interaction between FCDs and dislocations creates kinks on disclinations. As a result of this interaction, the screw dislocations will align along the kink. Unbinding of the dislocation loops will thus increase the kink density and may affect the FCD shape.

It has been recognized that formation of dislocation loops plays an important role in the smectic-nematic (SN) transition [49]. Helfrich [50] proposed that an increase in the dislocation loop density destroys the smectic order, and the unbinding of dislocation loops drives the SN-transition. In his model, the stability of dislocation loops is determined by its energy per unit length, *i.e*., line tension. When the temperature is increased, the line tension decreases and becomes negative above *T* = *T*_SN_. This leads to the spontaneous nucleation and proliferation of dislocation loops, and the decay of the smectic order is reflected by the temperature dependence of the layer compression modulus *B.* Benzekri *et al.* [51,52] showed that *B* decreases according to a power-law behavior with a critical exponent given by the Nelson-Toner model [53]. Using freeze-fracture transmission electron microscopy technique, Moreau *et al.* [54] showed that dislocation loop size indeed increases in the vicinity of the SN-transition for a lyotropic liquid crystal.

## 3. FCDs under Shear Flow

As Horn and Kleman presented [35], the FCD density increases by applying a shear flow. Their experimental observations suggest that non-equilibrium textural defects is additionally induced by the shear flow. Hence the smectic structures are strongly affected by the formation of non-equilibrium defects. In this section, we discuss the relation between the proliferation of dislocation loops and the FCD size and the shear rate [16].

**Figure 2.**
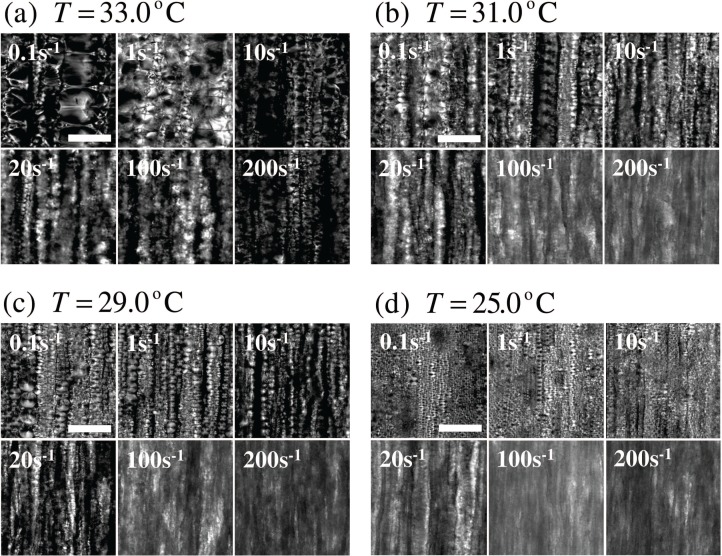
Polarized microscope images of the smectic phase under shear flow at different temperatures (**a**) *T* =33.0; (**b**) 31.0; (**c**) 29.0; (**d**) 25.0 °C and shear rates *γ̇* = 0.1, 1, 10, 20, 100, 200 s^−1^. The shear flow was applied along the vertical direction of the images which were taken just after the cessation of it. The scale bar corresponds to 100 μm.

Figure 2 shows polarized light microscope images of 8CB in the smectic phase under shear flow. These images were obtained immediately after applying different shear rates for 10 min for various temperatures ranging from the room temperature to *T*_SN_. The vertical and the depth directions correspond to the flow and the velocity gradient directions, respectively. It can be seen that strings of FCDs along the flow direction fill the space. We also note that the FCD size decreases with increasing the shear rate at each temperature. Under constant shear rate, on the other hand, the FCD size increases as the temperature approaches *T*_SN_.

**Figure 3.**
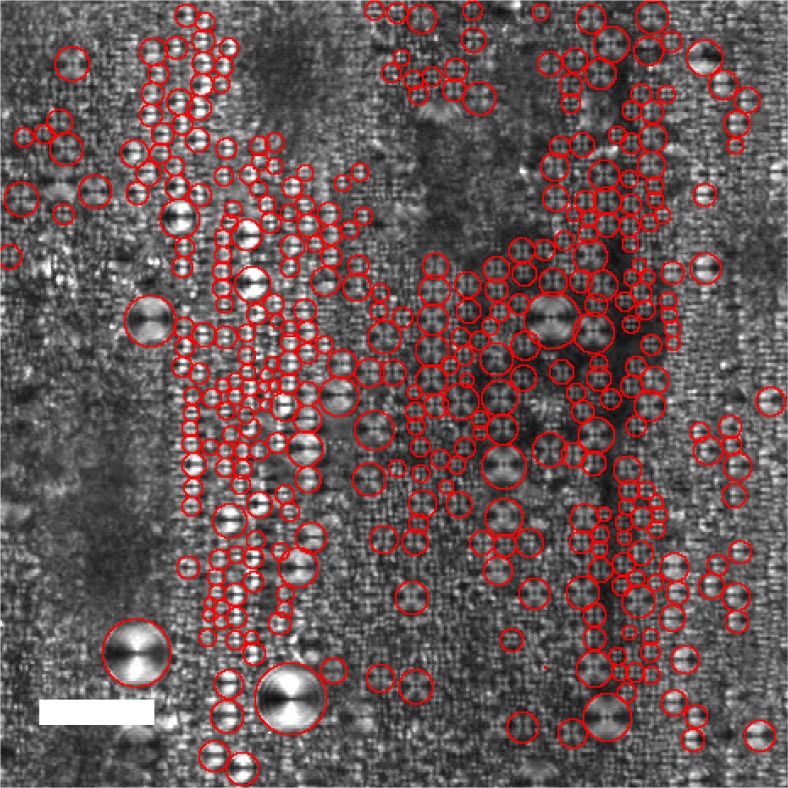
Typical microscope image used to estimate the FCD size at *T* =25.0 °C and *γ̇* = 0.1 s^−1^. The averaged diameter *L* of FCDs was estimated by tracing each FCD with a red circle as shown in the picture. The scale bar corresponds to 50 μm.

**Figure 4.**
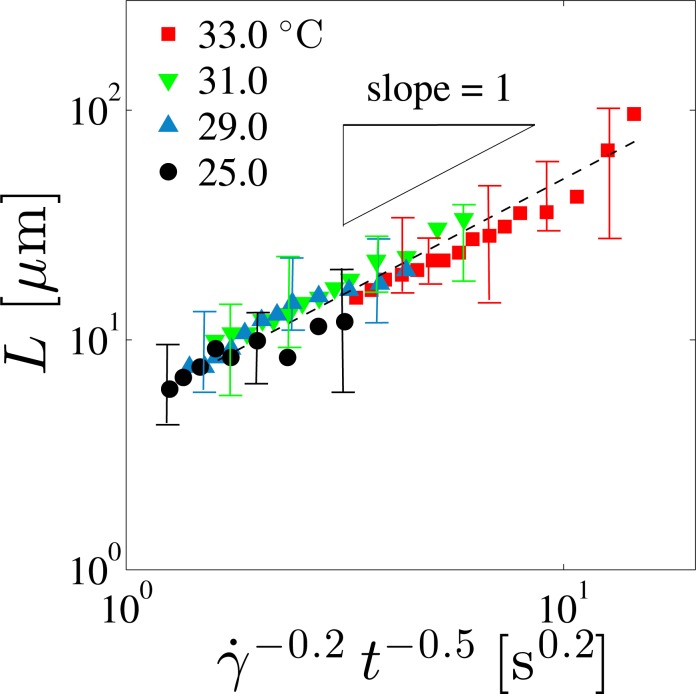
Log-log plot of the FCD size *L* as a function of the shear rate *γ̇* and the reduced temperature *t*. Different symbols correspond to different temperatures. The scaling variable is chosen to be *γ̇*^−0.2^*t*^−0.5^ so that all the data points fall onto a straight dashed line whose slope is unity.

To systematically study the effects of the temperature and shear rate on the FCD size, it is necessary to determine the mean diameter *L* of FCDs. A microscope image taken under the shear rate *γ̇*=0.1 s^−1^ is presented in Figure 3 as an example in order to show how to obtain *L*. As shown in the figure, the average of *L* can be estimated by tracing the distinguishable outlines of the FCDs with circles. Some FCDs are not used because their boundary contrast is too low. These FCDs are located either above or below the focus plane. We calculate *L* as the number average within the focus plane region. Close to the transition temperature *T*_SN_, one expects that the correlation length of the fluctuation and the dislocation size behave similarly to obey the scaling law in terms of the reduced temperature *t* = (*T*_SN_ − *T*)/*T*_SN_ [55,56]. From our experiment, we found that *L* depends on *t* and *γ̇* as *L* ∼ *γ̇*^−0.2^ (at each temperature) and *L* ∼ *t*^−0.5^ (at each shear rate), respectively. As presented in Figure 4, the FCD size *L* can be scaled by the combined variable *γ̇*^−0.2^*t*^−0.5^ .

Although there is no theory on the shear rate and/or temperature dependence of *L*, an analogous scaling relation with the same exponent was predicted for the defect spacing which varies as ∼ *γ̇*^−0.2^ [36,37]. Moreover, the average dislocation loop size diverges as ∼ *t*^−0.5^ according to the defect model by Helfrich [50]. We remind that dislocation loops are formed by pairs of edge and screw dislocations, and FCDs are linked by edge dislocations. Since the accumulation of the strain energy due to an increase in the defect density is the driving force for the FCD formation, the agreement of these exponents (0.2 and 0.5) suggests that the proliferation of dislocation loops controls the FCD size. Incidentally, it has been suggested that the non-equilibrium structural transition of lyotropic lamellar phase under shear is governed by the dislocation size [29].

## 4. Nonlinear Rheology of the Smectic Phase

It is known that the smectic phase exhibits both shear-thinning behavior and yield stress [24–26,28,35]. Although it has been anticipated that such rheological behavior is influenced by defects [41,42], we have further shown in the previous section that these structures are FCDs which originate from dislocation loops. In this section, we discuss how the presence of FCDs are reflected in the rheology of the smectic phase [16].

**Figure 5.**
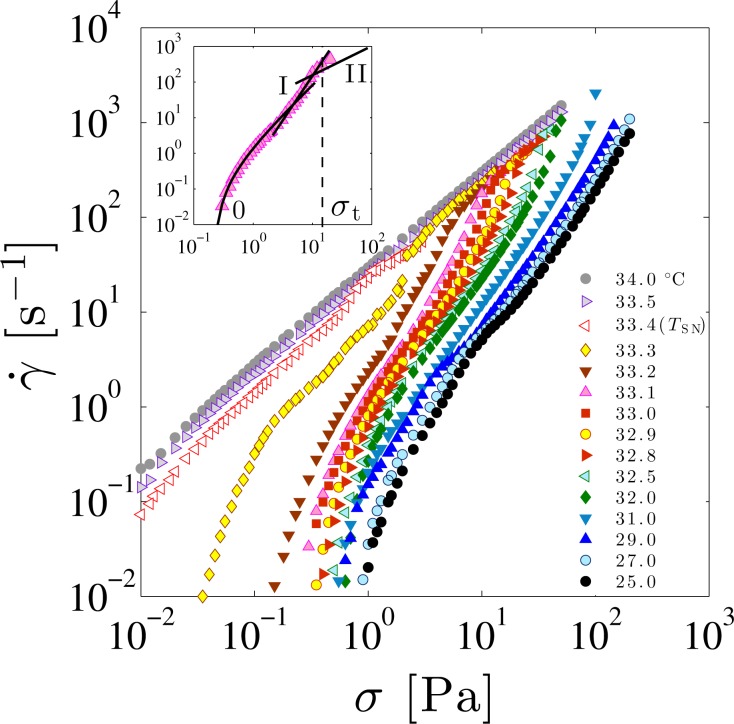
Log-log plot of the steady-steady shear rate *γ̇* as a function of the applied shear stress *σ* at different temperatures. The inset shows a typical curve (*T* =33.1 °C) divided into three regimes. Regime 0 is fitted by the Herschel-Bulkley model given by Equation (1); Regime I is fitted by the power-law behavior given by Equation (2); and Regime II corresponds to the Newtonian behavior. At the threshold stress *σ*_t_, the transition from Regime I to Regime II takes place. The SN-transition temperature *T*_SN_ is 33.4 °C.

Figure 5 shows the flow curves in the temperature range from 25.0 °C to 34.0 °C across the SN-transition temperature *T*_SN_ =33.4 °C. These flow curves are obtained by measuring the steady-state value of the shear rate *γ̇* when various values of the shear stress *σ* are applied. Within the power-law behavior *γ̇* ∼ *σ^m^*, *m* > 1 and *m* = 1 correspond to the shear-thinning and the Newtonian behaviors, respectively. Since *m* > 1 for *T* < *T*_SN_, the smectic phase exhibits a shear-thinning behavior.

It should be noted that the flow curves are not fully described by a single power-law since the slope in Figure 5 gradually changes as a function of the shear stress. Colby *et al.* [24] also observed a similar behavior of the flow curve. In order to discuss the nonlinear rheological response in more detail, we focus on the flow curve at *T* =25.0 °C as a typical example. First we realize that this flow curve reaches to a finite stress value when the shear rate is extrapolated to zero. This means that there is a yield stress *σ*_y_, below which the flow ceases. Previously, Horn and Kleman [35] or Colby *et al.* [25] reported that the smectic phase shows the yield stress. The flow curves for other temperatures also exhibit yield stress; it decreases at higher temperatures and vanishes at *T*_SN_. Furthermore, focusing on the high-shear flow curves near the transition point (e.g., *T* =33.0 °C), we see that the rheological behavior changes from shear-thinning to Newtonian at a specific shear stress. The corresponding threshold stress value *σ*_t_ required to become Newtonian shifts toward the lower value when approaching *T*_SN_. In the nematic phase at higher temperatures, only Newtonian behavior is observed.

To extract the temperature dependence of the power-law exponent and the yield stress, the flow curve was divided into three regions: shear-thinning region (Regime 0) showing the yield stress, shear-thinning region (Regime I) described by a power-law, and Newtonian region (Regime II). Notice that *σ*_t_ represents the boundary value between Regime I and Regime II. To estimate the yield stress in Regime 0, we use the empirical Herschel-Bulkley (HB) model

(1) $$\sigma =\sigma_{\text{y}}+A{\dot{\gamma }}^{n}$$

where *A* and *n* are parameters, and *σ*_y_ is the yield stress. This model has been frequently used to describe the non-Newtonian behaviors of yield stress fluids [57–59]. In addition to the HB model for Regime 0, Regime I was fitted with the power-law:

(2) $$\sigma =C{\dot{\gamma }}^{1/m}$$

Various quantities can be obtained by fitting these equations to the flow curve (see the inset of Figure 5).

**Figure 6.**
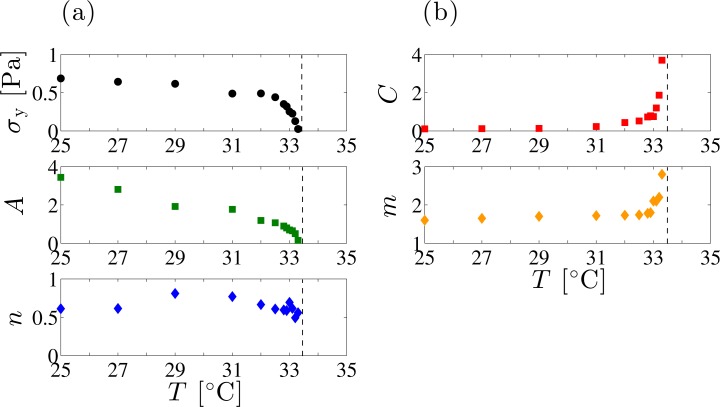
(**a**) The yield stress *σ*_y_, the pre-factor *A*, the exponent *n* in Equation (1), and (**b**) the pre-factor *C*, the shear-thinning exponent *m* in Equation (2) as a function of the temperature *T*. These values are obtained by the best fit in Figure 5 for each regime. Vertical dashed line indicates the SN-transition temperature; *T*_SN_ =33.4 °C.

The temperature dependence of each parameter is summarized in Figure 6. Whereas *σ*_y_ and *A* rapidly decrease and vanish at *T*_SN_, the exponent *n* does not show a simple temperature dependence. On the other hand, *C* and *m* are almost constant up to around *T* =32.0 °C and show a significant increase as *T*_SN_ is approached from below. The abrupt increase of *C* and *m* near *T*_SN_ indicates that the enhanced critical fluctuation or the proliferation of dislocation loops significantly affects the shear-thinning behavior. The shear-thinning exponent *m* ≈ 1.7 obtained at the low-temperature region coincides with the theoretically predicted exponent *m* = 5/3 by Kleman *et al.* [36,37].

As discussed in the previous section, the growth of dislocation loops causes the increase of the FCD size. Horn and Kleman employed a dimensional argument to relate the yield stress *σ*_y_ and the FCD size *L* by *σ*_y_ ∼ *K/L*^2^, where *K* is the bending modulus of the smectic phase [35]. Notice that the value of *K* is only weakly dependent on the temperature [60]. According to this relation, we see that a decrease of *σ*_y_ near *T*_SN_ corresponds to a rapid increase of *L*. Qualitatively, this temperature dependence of *L* agrees with the direct observations of FCDs in Figures 2 and 4. Hence we expect that the growth of dislocation loops influences the temperature dependence of *σ*_y_ through the increase of the FCD size. We shall further discuss the elasticity of FCDs in the next section.

**Figure 7.**
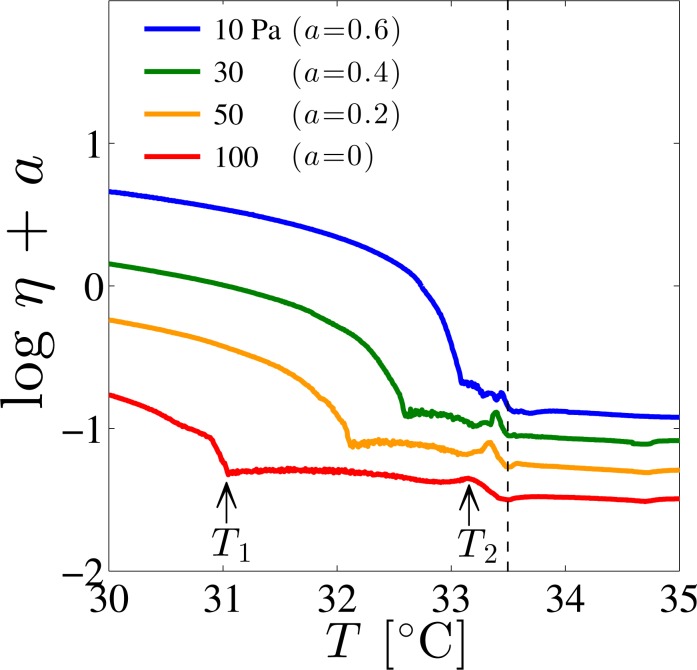
Shear viscosity *η* as a function of the temperature *T* obtained at different applied shear stress *σ* =10, 30, 50, 100 Pa. Here log *η* is shifted by a constant *a* in order to have a better visibility. Vertical dashed line indicates the SN-transition temperature; *T*_SN_ =33.4 °C.

Next we discuss the transition from shear-thinning (Regime I) to Newtonian behavior (Regime II) using the dynamic phase diagram. In addition to *σ*_t_ obtained from Figure 5, the temperature dependence of the viscosity *η* under constant shear stress was measured to construct non-equilibrium phase diagram. The temperature dependence of *η* measured at shear stress of *σ* =10, 30, 50, and 100 Pa is shown in Figure 7. Several results are found; (i) at low temperatures, *η* decreases as the temperature increases; (ii) above a certain temperature *T*_1_, *η* is almost constant; and (iii) at *T*_2_ slightly lower than *T*_SN_, a peak is observed. The two characteristic temperatures *T*_1_ and *T*_2_ shift toward lower values as the shear stress is increased.

**Figure 8.**
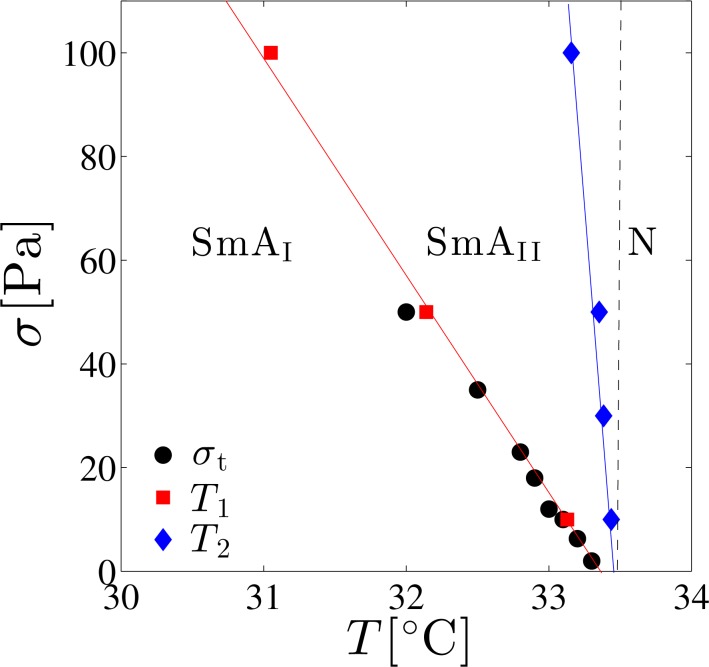
Dynamic phase diagram of 8CB under shear plotted against the temperature *T* and the applied shear stress *σ*. The smectic becomes Newtonian above *σ*_t_, whereas *T*_1_ and *T*_2_ are characteristic temperatures identified in Figure 7. The vertical dashed line indicates *T* = *T*_SN_. The lines of *σ*_t_ and *T*_1_ coincide with each other. “SmA_I_” and “SmA_II_” denote the smectic phases in Regime I and II, respectively, while “N” indicates the nematic phase.

The above results are summarized in the dynamic phase diagram presented in Figure 8. Here we find that the shear stress dependence of *T*_1_ and the temperature dependence of *σ*_t_ are almost identical to each other. Furthermore, both *T*_1_ and *T*_2_ change linearly with respect to *σ*. The temperature estimated by extrapolating *T*_1_ and *T*_2_ to zero shear stress coincides with *T*_SN_ at quiescent state, and the phase diagram can be divided into three regimes. Comparing with the flow curves of Figure 5 and the temperature dependence of *η* in Figure 7, we see that the low-temperature region (*T* ≤ *T*_1_) corresponds to the shear-thinning region (Regime I), while the temperature region *T*_1_ ≤ *T* ≤ *T*_2_ exhibits the Newtonian behavior (Regime II). For convenience, these two regions are denoted by “SmA_I_ phase” and “SmA_II_ phase”, respectively.

**Figure 9.**
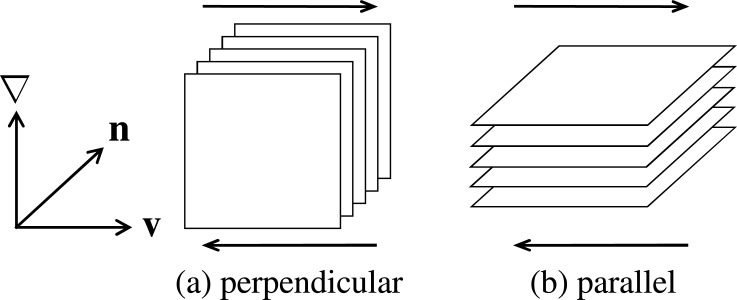
Schematic diagram of a smectic phase with perpendicular (left) and parallel (right) orientations under shear flow. ∇, **v** and **n** correspond to the flow gradient, flow and vorticity directions, respectively. The SmA_I_ phase consists of both perpendicular and parallel orientations, while only the perpendicular orientation appears in the SmA_II_ phase.

Previously, dynamic orientation diagram of the smectic phase was constructed with the use of rheo-physical methods such as small angle X-ray scattering under shear flow by Safinya *et al.* [27] and Panizza *et al.* [26], or rheo-dielectric measurement by Negita *et al.* [61]. Our dynamic phase diagram qualitatively agrees with their results. Although Safinya *et al.* [27] and Negita *et al.* [61] presented their diagrams as a function of the temperature and the shear rate, we also confirmed that our diagram roughly coincides with them by mapping the shear stress to the shear rate using the flow curves in Figure 5. As shown in Figure 9, it is known that two orientation states are possible in the smectic phase: perpendicular orientation in which the layer normal is perpendicular to both the velocity gradient and flow direction, and parallel orientation in which it is parallel to the velocity gradient direction [62,63].

Since the dynamic phase diagram obtained in Figure 8 and the orientation diagrams based on rheo-physical methods coincide, SmA_I_ phase is a mixture of the perpendicular and parallel orientations, or a leak structure for which layers are cylindrically rounded [26]. On the other hand, SmA_II_ phase consists of the perpendicular orientation. From this finding, we realize that the rheological behavior and the layer orientation are closely linked to each other. Furthermore, since *T*_2_ coincides with *T*_SN_ for sufficiently low-shear stress (*σ* =10 Pa), we anticipate that the peak in *η* reflects the precession motion of the monomers in the SN-transition [27,61]. The shift of *T*_2_ toward lower temperatures when subjected to high-shear stress implies that the SN-transition is induced by the shear flow. In addition to the proliferation of dislocation loops in thermal equilibrium state, the shear-induced SN-transition is due to the creation of non-equilibrium dislocation loops caused by the shear flow. The shear-induced SN-transition originates from the unbinding of dislocation loops which are created both under equilibrium and out-of-equilibrium conditions. A rich rheological behavior associated with the shear-induced layer orientation has been also found in the lyotropic lamellar phases [64–66].

## 5. Linear Viscoelasticity of the Smectic Phase

In this section, we discuss the influence of FCDs on the linear viscoelasticity of the smectic phase [17]. We also argue the physical origin of the elasticity of the smectic phase with FCDs. As described in the previous section, the dynamical smectic phase changes from the SmA_I_ phase to SmA_II_ phase as a function of the shear stress and temperature. Since the SmA_II_ phase exhibits only Newtonian behavior, we mainly concentrate on the viscoelasticity of the SmA_I_ phase.

In rheological measurements, the shear modulus *G* can be obtained by the ratio between the shear stress *σ* and the strain *γ* as *G* = *σ/γ*. On the other hand, *σ* is given by the product of the viscosity *η* and the shear rate *γ̇*, *i.e*., *σ* = *ηγ̇*. Viscoelastic materials exhibit both elastic and viscous responses which can be measured by applying oscillating strain with an angular frequency *ω* and an amplitude of *γ*_0_; *γ* = *γ*_0_ sin(*ωt*). The dynamic storage modulus *G′* and the loss modulus *G″* are determined by the following relation:

(3) $$\sigma =\gamma_{0}\left[G^{\prime }(\omega )\text{sin}(\omega t)+G^{\prime \prime }(\omega )\text{cos}(\omega t)\right]$$

In our experiment, all of the measurements were performed within the linear viscoelastic region which was confirmed by the strain sweep tests.

**Figure 10.**
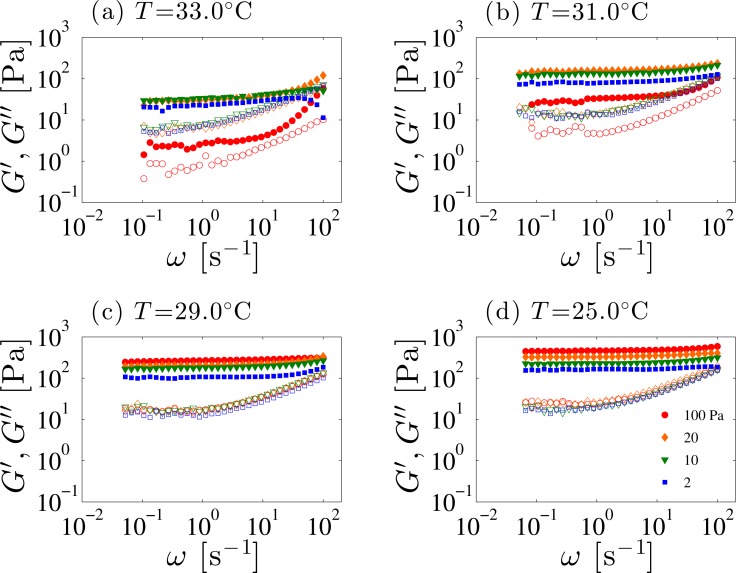
Log-log plot of the dynamic storage modulus *G′* and loss modulus *G″* as a function of the frequency *ω* at different temperatures. Filled and open symbols correspond to *G′* and *G″*, respectively. Different symbols shown in (**d**) represent the applied pre-shear stresses for which the steady states are obtained.

Figure 10 shows the frequency dependence of *G′* and *G″* measured after the system is subjected to a given pre-shear stress. For all temperatures, *G′* is always larger than *G″*. Moreover, a plateau region is observed in the low-frequency range, as Colby *et al.* also reported [24]. This solid-like viscoelastic behavior is strongly correlated with the defect density. In fact, Larson *et al.* [28] showed that both *G′* and *G″* decrease when the defects are removed by applying a large amplitude oscillatory shear. Hence the plateau modulus reflects the defect density.

**Figure 11.**
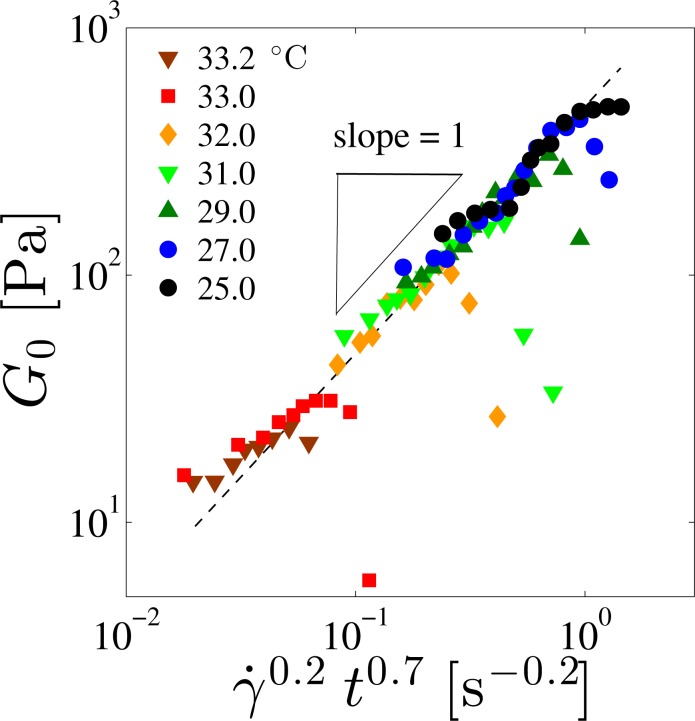
Log-log plot of the plateau shear modulus *G*_0_ as a function of the shear rate *γ̇* and the reduced temperature *t*. Different symbols correspond to different temperatures. The scaling variable is chosen as *γ̇*^0.2^*t*^0.7^ so that most of the data points fall onto a straight dashed line whose slope is unity.

Concerning the pre-shear stress dependence of the plateau value of *G′* (denoted as *G*_0_) at different temperatures, *G*_0_ becomes larger with increasing the pre-shear stress. However, as the temperature approaches to *T*_SN_ such as at *T* =33.0 °C, it decreases at high pre-shear stress and deviates from a simple scaling suggested in Figure 11. Comparing the pre-shear stress dependence of *G*_0_ to Figures 5 and 8, we notice that the shear stress value where *G*_0_ decreases is located near the boundary between the SmA_I_ and the SmA_II_ phases. Thus, the plateau value of *G′* reflects the defects associated with orientations of the layers. As discussed before, FCDs fill the SmA_I_ phase under shear flow, whereas they are not observed in the SmA_II_ phase. Hence, FCDs dominate the elasticity of the SmA_I_ phase. Once the orientation transition of the smectic layers takes place to become SmA_II_, the elasticity due to FCDs vanishes.

Similar to the temperature dependence of the yield stress, *G*_0_ also decreases as *T*_SN_ is approached. Since the FCD size *L* influences the shear modulus *G*, a similar scaling behavior found in Figure 4 is expected to hold. Here the value of *G′* at *ω* =0.1 s^−1^ was chosen as *G*_0_ which is plotted in Figure 11 as a function of the combined variable of *γ̇* and *t*. Adopting the result of Figure 5, the measured steady shear rate *γ̇* for each applied pre-shear stress *σ* can be used for the scaling plot. To obtain the scaling plot for *G*_0_, we first determined the power-law dependence *G*_0_ ∼ *γ̇*^0.2^ at each temperature. Then the power-law behavior of *G*_0_ as a function of *t* with an exponent 0.7 was extracted so that all the data points fall onto a straight line with a slope of unity, *i.e*., *G*_0_ ∼ *γ̇*^0.2^*t*^0.7^. Except for the data close to the border between the SmA_I_ and SmA_II_ phases, all *G*_0_ values fall on a straight line. Below, we discuss the physical meaning of these scaling behaviors for *G*_0_ and *L*.

When comparing the two scaling relations *G*_0_ ∼ *γ̇*^0.2^*t*^0.7^ and *L* ∼ *γ̇*^−0.2^*t*^−0.5^ obtained from independent measurements, it appears that *G*_0_ is almost inversely proportional to *L*, *G*_0_ ∼ 1/*L*, although the temperature exponent is slightly different. In order to satisfy this relation, the proportionality coefficient on the right hand side must have the dimension of surface tension, *i.e*., energy per unit area. For layered systems such as the smectic phase or the lamellar phase, de Gennes and van der Linden proposed an effective surface tension given by $\gamma_{\text{eff}}≃\sqrt{KB}$, where *K* and *B* are the bending and the compression moduli, respectively [55,67,68]. Here the numerical pre-factor is dropped. Thus the plateau shear modulus of the smectic phase *G*_0_ should obey the following relation:

(4) $$G_{0}=C^{\prime }\frac{\sqrt{KB}}{L}$$

where *C′* is the dimensionless proportionality coefficient. In the case of 8CB, it is known that *K* is almost constant, *K* = (5.2 ± 0.3) × 10^−12^ N [60], whereas *B* decreases with increasing temperature close to *T*_SN_. According to the experimental result of Benzekri *et al.*, the temperature dependence of *B* is given by *B* = (7.5 × 10^7^) × *t*^0.4±0.03^ Pa [51,52]. To verify the validity of Equation (4), we compare the value of *G*_0_*L* obtained from our experimental result and $\gamma_{\text{eff}}=\sqrt{KB}$ estimated from the literature, *i.e*., *G*_0_*L* = (4.56 × 10^−3^) × *t*^0.2^ Nm^−1^ and $\sqrt{KB}=\left(1.97\times 10^{-2}\right)\times t^{0.2}{\text{Nm}}^{-1}$. It is remarkable that both quantities scale as ∼ *t*^0.2^. This result implies that the temperature dependencies of *G*_0_ and *L* are related through the scaling *B* ∼ *t*^0.4^.

Based on our experimental result, we conclude that the physical origin of the elasticity in the smectic phase is the effective surface tension $\gamma_{\text{eff}}≃\sqrt{KB}$ of the FCDs. An analogous picture also holds for the elasticity of concentrated emulsions [69,70]. Furthermore, a similar relation to Equation (4) has also been observed for the onion phase in surfactant solutions which can be identified with FCD-II [71,72]. The proportionality coefficient *C′* for the onion phase is about *C′* ≈ 0.4 – 1.2, which is fairly close to *C′* =0.456/1.97 ≈ 0.23 obtained for the FCDs. The relatively small value of *C′* for 8CB may be due to the polydispersity of the FCD size. We mentioned before that FCD-I can be observed not only in the thermotropic smectic phase but also in the lyotropic lamellar phase, while FCD-II appears only in the lyotropic systems. Formation of these textures depend both on the bending and the Gaussian moduli. As shown in Figure 1, the main geometrical difference between FCD-I with toroidal shape and FCD-II with spherical shape is the sign of the Gaussian curvature. Therefore, the energy cost for the deformation of FCDs, as determined by $\sqrt{KB}$, dominates the elasticity in these two systems in spite of the geometrical difference. We expect that the origin of elasticity is a universal feature that is common to different layered systems.

Equation (4) for the elastic modulus *G*_0_ is different from the relation for the yield stress *σ*_y_ ∼ *K/L*^2^ predicted by Horn and Kleman [35]. It should be noted, however, that *G*_0_ in our case was measured within a linear regime, whereas the non-linear effect cannot be ignored in the estimation of yield stress. Generally, the shear modulus and the yield stress are not proportional to each other.

## 6. FCD Formation Induced by Shear Quench

In the previous sections, we mentioned that the FCDs induced by the unbinding of dislocations influence the viscoelasticity of the smectic phase. In this section, we explain our results in the non-equilibrium FCD formation when the system is subjected to shear stress-quench from high to low values [18]. Some studies on the FCD formation behavior under shear have been also reported [73,74].

**Figure 12.**
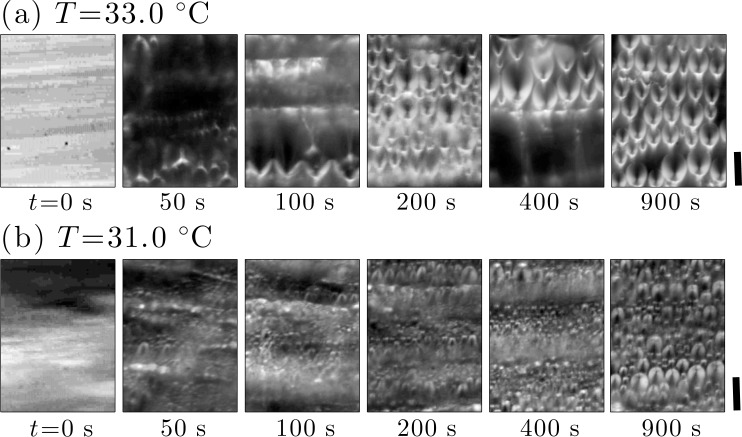
Time sequence of polarized images after quenching the system at (**a**) *T* = 33.0 °C and (**b**) *T* = 31.0 °C. Shear stress was quenched (**a**) from *σ* = 85 Pa to 0.1 Pa, and (**b**) from *σ* =85 Pa to 1.5 Pa, respectively. The horizontal direction is the flow direction. The FCDs are moving under the shear flow, and these images were picked out from the movies. The size of each image is 150 μm × 200 μm. The brightness of the image reflects the birefringence intensity. The scale bars correspond to 50 μm.

Microscope images in Figure 12a,b show the time sequences of snap shots after quenching the system from *σ* =85 to 0.1 Pa at *T* =33.0 °C, and from *σ* =85 to 1.5 Pa at *T* =31.0 °C, respectively. Comparing with the dynamic phase diagram in Figure 8, one sees that conditions of the stress-quench in (a) correspond to the FCD formation starting from the SmA_II_ phase, while (b) is the FCD growth inside the SmA_I_ phase. The brightness in the image represents the birefringence intensity.

The microscope image at *t* =0 s in Figure 12a is considerably bright because of the perpendicular orientation of the smectic layers [16,26,27]. After the stress-quench, the birefringence intensity quickly decays within a few seconds. Such a relaxation of the birefringence is caused by a flip of smectic layers from the perpendicular orientation to the parallel one. After the relaxation, a parabolic pattern appears around *t* =50 s, showing the appearance of FCDs whose number density increases as a function of time. At *t* =0 s in Figure 12b, on the other hand, the birefringence intensity is inhomogeneous. In later times, we see parabolic patterns as well as circular objects which are also FCDs with smaller sizes. There is a distribution of the FCD size, and the population of larger sizes increases with time. Around *t* =900 s in both cases, there are large FCDs aligned along the flow direction.

**Figure 13.**
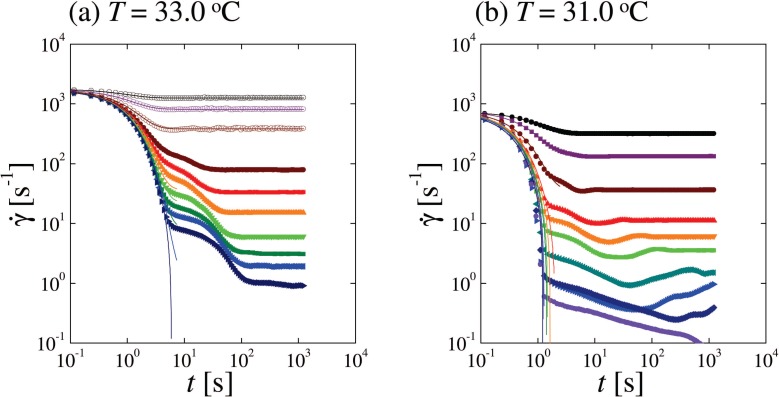
Log-log plot of the measured shear rate *γ̇* as a function of the elapsed time *t* after the stress-quench from *σ* =85 Pa to 60, 40, 20, 10, 7, 5, 3, 2, 1.5, 1 Pa (from upper to bottom) for (**a**) *T* =33.0 °C and (**b**) *T* =31.0 °C, respectively. Open and closed symbols correspond to the shear quench within the SmA_II_ phase and those from the SmA_II_ to the SmA_I_ phases, respectively. Data for (**b**) correspond to the stress-quench only within the SmA_I_ phase. Solid curves are the fits using a single exponential function with a characteristic time (first mode).

Figure 13 shows the measured shear rate *γ̇* as a function of time *t* for stress-quenches at the same temperatures as in Figure 12. In Figure 13a for *T* =33.0 °C, open symbols correspond to the stress-quench within the SmA_II_ phase, while closed symbols indicate the stress-quench from the SmA_II_ phase to the SmA_I_ phase. As drawn in the graph, the initial decay of *γ̇* can be fitted by a single exponential function which is called the first mode. On the other hand, when the system undergoes a non-equilibrium transition from the SmA_II_ to SmA_I_ phases, the shear rate exhibits a second decay mode as observed in Figure 13. In comparison with the microscope observations, we notice that the second mode appears when the formation of FCDs starts. As the terminal stress is decreased, the fraction of the second mode gradually increases.

The obtained relaxation time for the first mode shows a slowing down at *T*_SN_. Since the smectic layers in the SmA_II_ phase consist of a perpendicular orientation, the relaxation of the layer orientation accompanies a creation of edge dislocations before the FCD formation. It is known that the dislocations exhibit climb motion under shear stress [75,76]. The characteristic time for the climb motion of edge dislocation under stress is described by

(5) $$\tau_{\text{climb}}\sim \frac{d}{B\alpha b}$$

where *d* is the sample thickness, *α* the angle of the cone-plate shear cell, and *b* the mobility of the edge dislocation. Using typical values *d* ≈ 10 *μ*m, *B* ≈ 10^6^ Pa, *α* =0.017 rad, and *b* ≈ 10^−8^ m^2^s/kg, we obtain *τ*_climb_ ≃ 0.1 s, which roughly corresponds to the experimentally observed first mode. Hence the first relaxation can be attributed to the climb motion of edge dislocation, and the slowing down close to *T*_SN_ suggests that the climb motion is affected by the unbinding of the dislocation. Moreover, a similar slowing down is observed for the second mode. The validity of Equation (5) can be further checked by systematically changing *d* and *α*.

When the system is quenched within the SmA_I_ phase at *T* =31.0 °C as shown in Figure 13b, the shear rate shows anomalous behavior depending on the stress-quench depth. As the quench depth is increased, the two decay modes are observed similar to those for *T* =33.0 °C. However, there is a distinct third mode in which the shear rate increases (rather than decreases) after the double relaxation. Since this third mode corresponds to the time region when the alignment of FCDs takes place, it can be attributed to the formation of oily streaks. The third mode becomes slower when the temperature is decreased. Notice that oily streaks consist of FCDs connected by edge dislocations as depicted in Figure 1b. Hence the alignment of FCDs is affected by the dislocation unbinding [46]. Slowing down of these characteristic times in the vicinity of the SN-transition indicates that the dislocation unbinding dominates not only the SN-transition but also the dynamics of textural defects.

Here we shall qualitatively discuss the eccentricity of FCDs under shear flow. In Figure 12, elliptic FCDs can be seen with minor axis aligned along the flow direction. The eccentricity of the FCDs is caused by the shear since the FCDs at quiescent state are mostly circles as shown in Figure 3. When the shear stress is quenched, the layers will first disassemble along the velocity gradient direction, and may reconnect with the slightly tilted state to accommodate FCDs with the large Burger vectors [18]. Asymmetry of the FCDs along the vorticity direction would reflect the tilted layers in the FCDs. Moreover, the dynamic coupling between the dislocation loops and the shear flow may induce the distortion of the FCDs since the dislocation loops can adapt to the applied shear stress [38]. Kleman *et al.* [47] and Meyer *et al.* [48] indeed pointed out that the interaction of the FCDs with dislocations causes the distortion of the FCD shape. Detailed analysis on the eccentricity would clarify how the dislocation loops attribute to the FCD structure.

Finally, it is interesting to point out the similarity between the FCD (FCD-I) formation from the perpendicularly oriented layers and the onion (FCD-II) formation from the planar lamellae with parallel orientation. In our experiment, the double decay modes in the shear rate at fixed shear stress indicate that the viscosity increases with two steps. Such a behavior is also observed in the shear-induced onion formation at fixed shear rate [31,32]. Especially, the creation of dislocations prior to the FCD formation coincidences with the previous observation in the lyotropic system in which the increase of the defect density is essential for the onion formation [33]. Hence the proliferation of the dislocations plays an important role in the structural development from the planar layers for both thermotropic and lyotropic cases. A qualitatively similar time evolution of the viscosity in these systems suggests that the structural development dominated by defects is an universal mechanism in the layered systems.

## 7. Conclusions and Outlook

In this review article, we have discussed that the following properties are closely related to the unbinding of the dislocation loops; (i) scaling behavior of the FCD size; (ii) temperature dependence of the yield stress; (iii) shear-induced SN-transition; (iv) physical origin of elasticity; and (v) FCD formation from the SmA_II_ phase. In particular, we have clarified the physical origin of the elasticity by comparing the scaling relations for the FCD size and the shear modulus. This result indicates that the defects significantly affect the smectic rheology. Furthermore, the similarity in the rheological properties between FCDs and onions is a noteworthy consequence.

It should be noted that the origin of the nonlinear rheological behavior still remains to be clarified. Both the yield stress and the plateau shear modulus, commonly used to characterize the elasticity, depend on the FCD size. The shear modulus originates from the effective surface tension, whereas the source of the yield stress is poorly understood. Nonetheless, the estimated yield stress using an empirical relation *σ*_y_ ∼ *K/L*^2^ with *L* ≈ 10 – 10^2^ μm roughly coincides with our experimental observation in Figure 6. More detailed study will shed light on the yield stress in the smectic phase. By the same token, the shear-induced onion formation in lyotropic systems is strongly concerned with interactions of defects such as dislocations and oily streaks [30,33]. Understanding the origin of the nonlinearity in the smectic phase with defects will also lead to the elucidation of the shear-induced structural transition.

We expect that the concept of structural rheology is applicable not only to the smectic phase but also to other structured fluids [2,3,5,77–79]. One of the most interesting systems, which could be tackled by a similar concept, is the rheology of the blue phase in cholesteric nematic liquid crystals. In this phase, the interaction between disclinations generates the yield stress, and anomalous rheological behavior is expected depending on the type of disclination networks [80,81]. Especially, shear-induced breakup and reconnection of the disclination network may induce a new non-equilibrium structure, as observed under electric field [82]. Soft glassy nature of the amorphous blue phase due to the disordered disclination network is also an interesting issue related to the defect-mediated rheology. Melting of the amorphous blue phase due to shear can be explained by the proliferation of disclinations as in the lyotropic hexagonal phase [23].

Thanks to the development in visualization technology and microrheology method, better understanding of the stress response due to meso-scale structures has become possible in recent years [22,83–87]. Simultaneous measurements of the viscoelasticity and image acquisition will give us a time resolved spatial information under shear [77–79,88]. These technological developments are expected to contribute to the fundamental science of non-equilibrium soft matter in the future.
